# Are haematopoietic *stem cell* transplants *stem cell* transplants, is there *a threshold dose* of CD34-positive cells and how many are needed for rapid posttransplant granulocyte recovery?

**DOI:** 10.1038/s41375-023-01973-2

**Published:** 2023-07-20

**Authors:** Junren Chen, Robert Peter Gale, Yahui Feng, Yu Hu, Saibing Qi, Xueou Liu, Huaiping Zhu, Xiaowen Gong, Wei Zhang, Huilan Liu, Zimin Sun

**Affiliations:** 1 State Key Laboratory of Experimental Hematology, National Clinical Research Center for Blood Diseases, Haihe Laboratory of Cell Ecosystem, Institute of Hematology & Blood Diseases Hospital, Chinese Academy of Medical Sciences & Peking Union Medical College, Tianjin, China; 2 Tianjin Institutes of Health Science, Tianjin, China; 3 Centre for Haematology, Department of Immunology and Inflammation, Imperial College of Science, Technology and Medicine, London, UK; 4 Department of Hematology, The First Affiliated Hospital of University of Science and Technology of China, Hefei, China; 5 Blood and Cell Therapy Institute, Division of Life Sciences and Medicine, Anhui Provincial Key Laboratory of Blood Research and Applications, University of Science and Technology of China, Hefei, China

**Keywords:** Haematopoietic stem cells, Stem-cell research

*One of my greatest pleasures in writing has come from the thought that perhaps my work might annoy someone of comfortably pretentious position. Then comes the saddening realization that such people rarely read*.

John Kenneth Galbraith

## Introduction

It is widely-believed recovery of bone marrow function after a haematopoietic cell transplant arises from pluripotent haematopoietic *stem cells* (HSCs; hence haematopoietic *stem cell* transplant) [1], that numbers of CD34-positive cells in a graft are an accurate proxy for numbers of HSCs [2, 3], that dose of CD34-positive cells should be quantified by body weight and that there is a *threshold dose* of CD34-positive cells required for successful posttransplant bone marrow recovery [4–9].

In this *Perspective* we argue several or all of these commonly held notions are wrong. We consider biological plausibility, experimental data in mice and humans and advanced statistical analyses of a dataset of recipients of cord blood cell transplants for leukaemia. Our conclusions have important implications for transplant practices.

## Experimental data

### Can we accurately identify human HSCs and are they CD34-positive?

We start emphasizing currently there is no accurate nor precise way to identify human haematopoietic *stem* cells (HSCs) [10–12]. Data from transplanting human bone marrow into immune-deficient mice suggest 10E-6 to 10E-7 of mononuclear cells might be HSCs, a frequency which might be higher in human umbilical cord blood, lower in blood cells and still lower in so-called *mobilised* blood cells [13]. Because most mononuclear cells are not HSCs many people have focused on measuring CD34-positive cells assuming human HSCs are CD34-positive. Lacking an accurate and precise assay for humans HSCs [14–16] this is, of course, conjecture. Moreover, in mice quiescent HSCs are CD34-negative and express CD34 only after they begin to divide [17]. Whether this is so in humans is unknown. Other data suggest a constantly-changing phenotype of human HSCs [11]. Regardless, most CD34-positive cells in bone marrow, blood and umbilical cord blood are not HSCs [14]. Because almost all CD34-positive cells in a graft are not HSCs estimating numbers of HSCs based on numbers of CD34-positive cells must be imprecise. For instance, if we assume the ratio of HSCs to CD34-positive cells is 1 to 5000 in umbilical cord blood a graft containing 40 × 10E+5 CD34-positive cells could have 10 percent variation in numbers of HSCs because of Poisson noise.

### What cells are responsible for posttransplant recovery of bone marrow function

Another complexity is that we don’t know which haematopoietic cell(s) restore posttransplant bone marrow function. Data in mice indicate many different cells including many which are not HSCs contribute to short- and long-term posttransplant bone marrow recovery [18–20]. Data in humans are largely consistent with data in mice where short- and long-term bone marrow recovery are driven by different cell types [21–23]. Even within the phenotypically most primitive HSC pool individual *stem cells* can have very different self-renewal potentials and different contributions to bone marrow recovery [24–30]. Namely, not all HSCs contribute equally to sustained multi-lineage haematopoiesis and most show biased differentiation towards specific haematopoietic lineages.

## Clinical considerations

Considering the above we are left with 3 currently insoluble problems. 1st, we cannot accurately and precisely identify human HSCs. 2nd, we do not know which cells in a graft (HSCs, others or most likely a combination) are responsible for short- and long-term posttransplant recovery of bone marrow function. 3rd, it is highly likely residual recipient haematopoietic cells contribute in whole or part to posttransplant bone marrow recovery [31]; this is especially likely after less intensive pretransplant conditioning regimens (reduced-intensity [RIC] and non-myelo-ablative) but recovery of endogenous haematopoiesis is still plausible even after high-dose total body radiation without a transplant [32, 33] .

Despite these uncertainties transplant physicians feel compelled to address 2 questions: (1) how to derive a measure for haematopoietic potential of a graft using the number of CD34-positive cells despite the caveats we discussed; and (2) whether there is a *threshold dose* needed for successful posttransplant bone marrow recovery.

Although numbers of CD34-positive cells in a graft and ability to restore posttransplant bone marrow function are dissimilar, provided the two maintain a relatively predictable ratio CD34-positive cell dose might still be a useful surrogate. This raises the question of how numbers of CD34-positive cells can be converted to a dose. Presently, CD34-positive cell dose is quantified by body weight [4–9, 34]. The biological justification for this widely-adopted practice is unclear: In the graft we are dealing with some cells with substantial proliferative potential, not a drug that is stoichiometrically metabolised by the liver or excreted by the kidneys. Mammals come in various sizes from a mouse (20 g) to humans (70 kg) to elephants (6000 kg). Although an elephant has many more cells than a mouse (3 × 10E+9 *versus* 10E+15) the haematopoietic systems in both arise from one or a few HSCs. A human gaining 20 kg does not suddenly have more cells (just bigger fat cells) and his or her blood volume certainly would not suddenly increase by 30 percent because blood volume correlates better with lean body mass than with body weight, body mass index or body surface area across both sexes at all Tanner stages [35].^1^ Does such a person need 30 percent more CD34-positive cells to recover posttransplant bone marrow function were we to calculate this by body weight? Obviously no [36, 37].

### Is there a required threshold dose for CD34-positive cells?

**Fig. 1:**
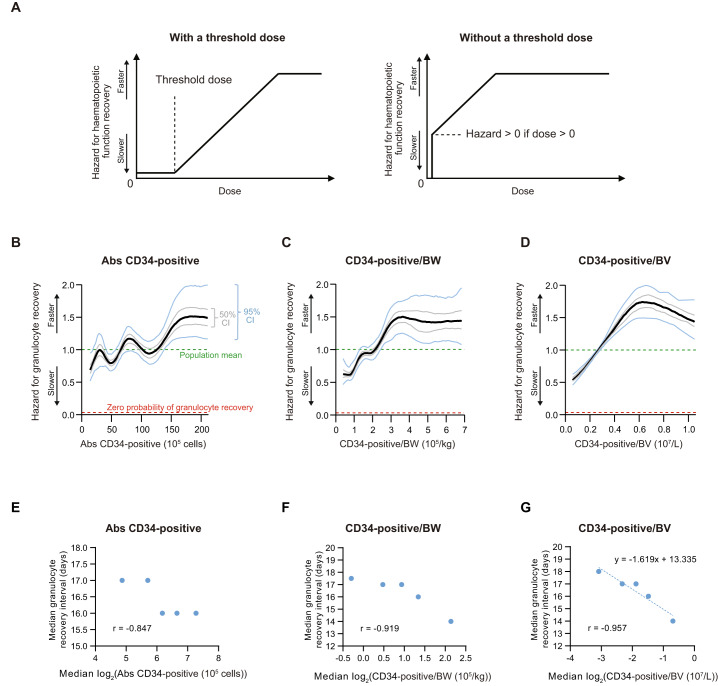
Dose response of CD34-positive cells. **A** Hazard functions for haematopoietic function recovery under two contrasting scenarios: with or without a *threshold dose*. Hazard functions of Abs CD34-positive (**B**), CD34-positive/BW (**C**) and CD34-positive/BV (**D**) for granulocyte recovery in the analysed umbilical cord blood cell transplant data (*N* = 619). Hazard is calculated with respect to the population mean. “Hazard = 0.5” means that the instantaneous recovery rate (from day 1 posttransplant to infinity) is half-magnitude compared to the population mean. “Hazard = 0” means zero probability of granulocyte recovery. **E**, **F**, **G** Relationship between CD34-positive cell dose and interval to granulocyte recovery: Abs CD34-positive (**E**), CD34-positive/BW (**F**) and CD34-positive/BV (**G**) in the analysed umbilical cord blood cell transplant data (*N* = 609; patients who died before granulocyte recovery are excluded from this analysis). In each panel, all the patients are divided into 5 quintiles according to the panel’s respective measure of CD34-positive cell dose. Each dot summarises one quintile, with its *x* and *y* coordinates representing the median CD34-positive cell dose and the median interval to granulocyte recovery of the quintile. *Abs* absolute, *BV* blood volume, *BW* body weight.

We further argue it is biologically implausible there is a *threshold dose* of CD34-positive cells for successful posttransplant bone marrow recovery. If there is a *threshold dose* the hazard function for recovery of bone marrow function is expected to be zero until the dose, however quantified, exceeds the threshold. Conversely, if there is no *threshold dose*, the hazard function would *continuously* increase unless the dose is zero (Fig. 1A). Previous research that interrogated the relationship between CD34-positive cell dose and posttransplant bone marrow recovery uniformly analysed dose by discretising it into multiple classes (Table 1) [38–47]. This approach cannot uncover the shape of the dose-response curve for CD34-positive cell dose *versus* haematopoietic function recovery and therefore is not suitable for answering the question whether there is a *threshold dose* of CD34-positive cells [48]. Previous studies also reported contradictory data on correlations between CD34-positive cell dose and various transplant endpoints such as survival [38–47]. This likely reflects confounding co-variates including sex, age, disease, disease state, pre- and posttransplant conditioning and immune-suppression regimens, histo-compatibility between donor and recipient, graft-type, development of graft-*versus*-host disease, interstitial pneumonia and others.

**Table 1:** Impact of low CD34-positive cell dose on posttransplant granulocyte recovery in several large-cohort studies.

Reference	Graft-type	Donor-type	*N*	Definition of low dose (/kg)	Lowest dose (/kg)	Impact of low dose on granulocyte recovery
[38]	Mobilised blood	Self	508	< 3.00 × 10E+6	1.90 × 10E+6	Adverse
[39]	Mobilised blood	Unrelated (59% HLA-identical)	611	≤ 3.8 × 10E+6	0.4 × 10E+6	Adverse
[40]	Mobilised blood	HLA-identical sibs	370	< 4 × 10E+6	–	Adverse
[40]	Mobilised blood	Unrelated (76% HLA-identical)	687	< 6 × 10E+6	–	NS
[41]	Mobilised blood	Various	705	< 1.08 × 10E+7	8.3 × 10E+5	Adverse
[43]	Mobilised blood	HLA-haplotype matched	< 348^a^	≤ 1.01 × 10E+6	1.3 × 10E+5	Adverse
[44]	Mobilised blood	Related (62% HLA-identical)	2919	< 1 × 10E+6	–	Adverse
[46]	Mobilised blood	Mostly HLA-identical sibs	851	< 4.5 × 10E+6	6.5 × 10E+5	Adverse
[47]	Mobilised blood	HLA-identical sibs	377	< 5.0 × 10E+6	1.3 × 10E+6	Adverse
[42]	Umbilical cord blood	Unrelated (2% HLA-identical)	306	< 5 × 10E+4	1.5 × 10E+4	Adverse
[45]	Umbilical cord blood	Unrelated ( ≈ 5% HLA-identical)	1351	< 6.1 × 10E+4	–	Adverse

– not available, *NS* not significant.

^a^This study included both G-CSF-primed bone marrow cell transplants and mobilised blood cell transplants. The exact number of blood cell transplants was not stated.

Consensus guidelines suggest a *threshold dose* of CD34-positive cell dose ranging from 1.5 × 10E+5/kg for umbilical cord blood cell grafts to 4 or 5 × 10E+6/kg for mobilised blood cell grafts [4–9]. Applying this recommendation suggests only 4% of the US cord blood inventory is suitable for single-unit transplants for adults [49]. Theoretically, however, even one HSC is capable of restoring posttransplant bone marrow function given sufficient time (provided we can keep the recipient alive for a prolonged interval) [13, 37]. Data in mice indicate some HSC clones are highly efficient in restoring long-term bone marrow function [50]. Moreover, we reported recovery of bone marrow function in a person exposed to acute extremely high-dose and -dose-rate total body radiation without a transplant [32, 33], indicating the jargon of *myelo-ablative* pretransplant conditioning is wrong. Because radiation killing of cells is stochastic it is nearly impossible ionising radiation could kill every HSC without killing the person.

### Clinical data

To help resolve these challenges and controversies and despite our reservations we interrogated data from 619 consecutive subjects with acute leukaemia receiving a single-unit umbilical cord blood cell transplant (details in Supplement Table 1; Supplement Fig. 1). We chose umbilical cord blood cell transplants because an insufficient CD34-positive cell dose is often cited as the reason to exclude potential recipients (mostly adults) [51] and because umbilical cord blood likely has the highest fraction of HSCs amongst CD34-positive cells [13]. Almost one-half of subjects in our cohort were > 16 years. The lowest, 5th-, 10th- and 25th-percentile values of CD34-positive cells *per* kg of recipient body weight were 0.17, 0.60, 0.83 and 1.28 × 10E+5/kg. Namely, the lowest CD34-positive cell dose was almost one-tenth of the *threshold dose* according to consensus guidelines. Additionally, the CD34-positive cells were quantified at a central laboratory (Supplement Methods) avoiding non-standardized CD34-positive cell quantification confounding many studies. Our dataset allowed us to *pressure-test* the idea of *threshold dose* for successful posttransplant bone marrow recovery.

We considered 3 expressions of CD34-positive cell dose: (1) absolute numbers of CD34-positive cells (Abs CD34-positive); (2) numbers of CD34-positive cells *per* kg of recipient body weight (CD34-positive/BW); and (3) numbers of CD34-positive cells *per* litre of recipient blood volume (CD34-positive/BV; “BV” stands for blood volume), with blood volume estimated as described [35].

Our focus was on granulocyte recovery because analyses of RBC and platelet recovery are confounded by pre- and posttransplant transfusions, granulocytes are the most short-lived cells and granulocyte recovery is most closely correlated with early posttransplant therapy-related mortality. Analyses of survival and other transplant endpoints are tangential to our primary concern because of confounders such as graft-*versus*-host disease and leukaemia recurrence. For each measure of CD34-positive cell dose we calculated two concordance values: (1) concordance with interval to granulocyte recovery (equivalent to the area under the receiver-operating characteristic curve) [52]; and (2) concordance with granulocyte recovery within 21 days by fitting a logistic regression model [53]. Among the three expressions of CD34-positive cell dose, CD34-positive/BV had the highest concordance with granulocyte recovery (Table 2), especially in instances with “extreme” ratios ( < 15th- or >85th-percentile values) of lean body mass to body weight. Results were similar when subjects receiving pretransplant radiation and/or posttransplant methotrexate were censored (Supplement Table 2).

**Table 2:** Concordance of CD34-positive cell dose with granulocyte recovery in the analysed umbilical cord blood cell transplant data.

	*N*	Absolute CD34-positive	CD34-positive per BW	CD34-positive per BV
**Concordance with interval to recovery**				
All cases	619	0.548	0.607	0.607
“Extreme” LBM/BW ratio^a^	186	0.524	0.606	0.610
“Normal” LBM/BW ratio^a^	433	0.557	0.609	0.609
**Concordance with successful recovery by d 21 posttransplant**^b^				
All cases	609	0.625	0.654	0.672
“Extreme” LBM/BW ratio^a^	183	0.575	0.592	0.636
“Normal” LBM/BW ratio^a^	426	0.640	0.698	0.702

*BV* blood volume, *BW* body weight, *LBM* lean body mass.

^a^“Extreme”, < 15th- or > 85th-percentile values; “normal“, other cases.

^b^Patients who died before granulocyte recovery were excluded from this analysis.

We used a Bayesian Cox regression model with restricted cubic splines to estimate the non-linear CD34-positive cell dose effect (Supplement Methods) and roughness penalty minimization and Markov chain Monte Carlo to estimate confidence intervals of the dose-response curves [54, 55]. We found the hazard function for granulocyte recovery was erratic when CD34-positive cell dose was quantified as absolute numbers of CD34-positive cells (Fig. 1B). In contrast, the hazard function of CD34-positive cell dose *per* kilogram of recipient body weight plateaued at ≈ 1.5 once CD34-positive/BW was > 3 × 10E+5/kg, and there was no threshold value below which the hazard function abruptly dropped to zero (Fig. 1C). The hazard function of CD34-positive cell dose *per* litre of recipient blood volume plateaued at ≈ 1.5 when CD34-positive /BV was > 0.5 × 10E+7/L and the hazard remained ≈ 0.5 even when CD34-positive/BV dropped to 0.05 × 10E+7/L, the 2.5th-percentile value in this study cohort (Fig. 1D). Results were similar when subjects receiving pretransplant radiation and/or posttransplant methotrexate were censored (Supplement Fig. 2).

Next, we divided the subjects into quintiles according to CD34-positive cell dose and for each quintile calculated median log_2_(CD34-positive cell dose) *versus* interval to granulocyte recovery. Quintile median log_2_(dose) and quintile median interval to granulocyte recovery correlated the best when dose was quantified *per* blood volume (*r* = –0.96 (CD34-positive/BV) *versus* –0.85 (Abs CD34-positive) and –0.92 (CD34-positive/BW); Fig. 1E–G). Analysis of the linear regression coefficients of quintile median interval to granulocyte recovery *versus* quintile log_2_(CD34-positive/BV) suggests the number of granulocytes in the blood doubled every 1.6 days (Fig. 1G). Even in the lowest quintile of CD34-positive/BV dose blood concentrations of NK-, CD8-positive T-, CD4-positive T- and B normalised within 1 year posttransplant, and all the quintiles had similar cumulative incidences of relapse and survival (Supplement Figs. 3 & 4). In multi-variate Cox regression analysis of granulocyte recovery CD34-positive/BV (HR = 1.89 per 10E+7/L [1.42, 2.51]; adjusted *P* < 0.001) was independently correlated with cumulative incidence of granulocyte recovery (Supplement Fig. 5).

## Discussion

We review biological considerations and experimental data indicating cells responsible for posttransplant bone marrow recovery in humans cannot be accurately nor precisely quantified. We also argue why numbers or dose of CD34-positive cells in a graft cannot be an accurate estimate of numbers of cells responsible for posttransplant bone marrow recovery. Nevertheless, given the several studies reporting a correlation between numbers of CD34-positive cells and posttransplant recovery of bone marrow function we used new statistical methods to interrogate a large dataset of umbilical cord blood cell transplants to prove the non-linear CD34-positive cell dose effect and show there is no *threshold dose* needed for posttransplant recovery of bone marrow function. This observation has important clinical implications which may make more people eligible recipients of a haematopoietic cell transplant, especially an umbilical cord blood cell transplant, and reduced the perceived need for repeated leukaphereses to obtain *sufficient* numbers of CD34-positive cells from donors.

Despite no *threshold dose* for posttransplant granulocyte recovery, physicians may want an estimate of the speed with which this occurs. To accomplish this we found CD34-positive cell dose should be calculated based on recipient blood volume, not body weight. Our analyses indicate a near-perfect linear relationship between log_2_(CD34-positive/BV) and interval to granulocyte recovery. Using this metric the expansion of granulocytes is close to exponential immediately posttransplant. Because of the rarity of HSCs in a haematopoietic cell graft the observed correlation between numbers or dose of CD34-positive cells and rate of posttransplant granulocyte recovery is consistent with the hypothesis precursor and progenitor cells operate in this setting rather than HSCs. (Contradictory data have been reported using gene marker studies [56].) An online calculator for CD34-positive cell dose by recipient blood volume is available at https://skirt-calculator.shinyapps.io/CD34-positive_Cell_Dose_Calculator/.

Our study has limitations. 1st, we suggest our conclusions apply to other graft types such as bone marrow and blood but this needs validation. 2nd, our analyses of the clinical dataset were retrospective and potentially biased. 3rd, although we studied a range of CD34-positive cell doses none was < 0.02 × 10E+7/L or < 0.17 × 10E+5/kg.

In our *Perspective*, we challenge the notion haematopoietic cell transplants are proved to be stem cell transplants. We also challenge current thinking and practices regarding whether CD34-positive cell dose in a graft should be used as a proxy for predicting short- and long-term posttransplant bone marrow recovery and, if there is no alternative, how CD34-positive cell dose should be quantified. Lastly, we show there is no *threshold dose* of CD34-positive cells (regardless of how dose is quantified) for successful posttransplant recovery of bone marrow function.

Physicians are conservative and reluctant to change long-established practices regardless of their validity. Our conclusions challenge several *sacred cows* and we anticipate resistance to accepting them. It is important to recall many firmly-held medical practices are later proved ineffective or even harmful. These situations are termed *medical reversals* and there are many examples reviewed in recent biomedical literature [57–59]. We look forward to validation of our conclusions.

### Supplementary information

SUPPLEMENTAL MATERIAL

## Data Availability

Clinical data are available upon reasonable request to the corresponding authours.

## Footnotes

1 For example, lean body mass (in kg) of a male or a prepubescent female can be estimated by 0.407 × body-weight (in kg) + 26.7 × body height (in m) – 19.2. Each kg increase of lean body mass translates to 83.8 ml increase of blood volume [35]. A 175 cm tall and 67 kg weight adult male has a lean body mass of 54.8 kg and a blood volume of 4.59 L. After gaining 20 kg his lean body mass increases to 62.9 kg and his blood volume to 5.27 L, a 15 percent increase.
