# Correction: Are haematopoietic *stem cell* transplants *stem cell* transplants, is there *a threshold dose* of CD34-positive cells and how many are needed for rapid posttransplant granulocyte recovery?

**DOI:** 10.1038/s41375-023-02012-w

**Published:** 2023-08-30

**Authors:** Junren Chen, Robert Peter Gale, Yahui Feng, Yu Hu, Saibing Qi, Xueou Liu, Huaiping Zhu, Xiaowen Gong, Wei Zhang, Huilan Liu, Zimin Sun

**Affiliations:** 1 State Key Laboratory of Experimental Hematology, National Clinical Research Center for Blood Diseases, Haihe Laboratory of Cell Ecosystem, Institute of Hematology & Blood Diseases Hospital, Chinese Academy of Medical Sciences & Peking Union Medical College, Tianjin, China; 2 Tianjin Institutes of Health Science, Tianjin, China; 3 Centre for Haematology, Department of Immunology and Inflammation, Imperial College of Science, Technology and Medicine, London, UK; 4 Department of Hematology, The First Affiliated Hospital of University of Science and Technology of China, Hefei, China; 5 Blood and Cell Therapy Institute, Division of Life Sciences and Medicine, Anhui Provincial Key Laboratory of Blood Research and Applications, University of Science and Technology of China, Hefei, China

**Keywords:** Haematopoietic stem cells, Stem-cell research

Correction to: *Leukemia* 10.1038/s41375-023-01973-2, published online 20 July 2023

In the original published online version, “<1.08 × 10E+7” in row #5 of Table 1 (ref. 41) was misquoted as “<1.82 × 10E+7”. Table 1 should have appeared as shown below. The original article has been corrected.

**Table 1:** Impact of low CD34-positive cell dose on posttransplant granulocyte recovery in several large-cohort studies.

Reference	Graft-type	Donor-type	*N*	Definition of low dose (/kg)	Lowest dose (/kg)	Impact of low dose on granulocyte recovery
[38]	Mobilised blood	Self	508	<3.00 × 10E+6	1.90 × 10E+6	Adverse
[39]	Mobilised blood	Unrelated (59% HLA-identical)	611	≤3.8 × 10E+6	0.4 × 10E+6	Adverse
[40]	Mobilised blood	HLA-identical sibs	370	<4 × 10E+6	–	Adverse
[40]	Mobilised blood	Unrelated (76% HLA-identical)	687	<6 × 10E+6	–	NS
[41]	Mobilised blood	Various	705	<1.08 × 10E+7	8.3 × 10E+5	Adverse
[43]	Mobilised blood	HLA-haplotype matched	<348^a^	≤1.01 × 10E+6	1.3 × 10E+5	Adverse
[44]	Mobilised blood	Related (62% HLA-identical)	2919	<1 × 10E+6	–	Adverse
[46]	Mobilised blood	Mostly HLA-identical sibs	851	<4.5 × 10E+6	6.5 × 10E+5	Adverse
[47]	Mobilised blood	HLA-identical sibs	377	<5.0 × 10E+6	1.3 × 10E+6	Adverse
[42]	Umbilical cord blood	Unrelated (2% HLA-identical)	306	<5 × 10E+4	1.5 × 10E+4	Adverse
[45]	Umbilical cord blood	Unrelated ( ≈ 5% HLA-identical)	1351	<6.1 × 10E+4	–	Adverse

– not available, *NS* not significant.

^a^This study included both G-CSF-primed bone marrow cell transplants and mobilised blood cell transplants. The exact number of blood cell transplants was not stated.

