# Supplementary material for: Are haematopoietic stem cell transplants stem cell transplants, is there a threshold dose of CD34-positive cells and how many are needed for rapid posttransplant granulocyte recovery?
Source: Leukemia. 2023 Jul 20;37(10):1963–8. doi: 10.1038/s41375-023-01973-2 (PMC10539175; doi:10.1038/s41375-023-01973-2)
Supplement: Supplementary file 1 — SUPPLEMENTAL MATERIAL [file 41375_2023_1973_MOESM1_ESM.pdf]

SUPPLEMENT MATERIAL

Supplement Methods

Umbilical cord blood cell transplant data

We interrogated data from 619 consecutive subjects with acute leukaemia receiving a single-unit umbilical cord blood cell transplants from 1 January 2015 to 26 December 2020 at the First Affiliated Hospital of University of Science and Technology of China, Hefei, China (**Supplement Table 1** and **Supplement Figure 1**). There were 343 males (55%). Median age was 13.6 years (Interquartile range [IQR], 7.2 – 29.6 years), and 272 (44%) patients were adults (age > 16). 339 subjects had AML (55%) and 270 (44%), ALL. Acute myeloid leukaemia (AML) risk was defined according to the 2022 ELN risk classification [1]. High-risk acute lymphoblastic leukaemia (ALL) was defined according to the NCCN Clinical Practice Guideline for ALL, version 1.2022 [2]. Median interval from diagnosis to transplant was 7.6 months (IQR, 5.2 – 14.9 months). Pretransplant conditioning was with busulfan and cyclophosphamide with or without other drugs such as fludarabine, cytarabine, decitabine, carmustine, cladribine or chidamide (N = 576 (93%)) or with radiation and cyclophosphamide with or without other drugs such as fludarabine, cytarabine, decitabine or carmustine (N = 43 (7%)). No patient was administered antithymocyte globulin. Posttransplant immune suppression was with cyclosporine and mycophenolate mofetil without (N = 612 (99%)) or with methotrexate (N = 7 (1%)).

All the subjects received one unit of umbilical cord blood cells. Frozen cord blood units were obtained from provincial-level cord blood banks in Beijing,

Guangdong, Shandong, Shanghai, Sichuan, Tianjin or Zhejiang, China and thawed immediately before infusion at 37 °C for 2 min. A 0.1-mL aliquot was taken from each thawed cord blood unit and assayed in a central laboratory at FAHUSTC to quantify numbers and concentration of CD34-positive cells using the dual-platform ISHAGE protocol [3]. Percentage of CD34-positive cells was quantified by multi-parameter flow cytometry (MPFC) analysis of 10E+5 cells (Navios; Beckman Coulter, Brea, CA, USA) with antibodies BD™ CD34 APC and BD™ CD45 PerCP (BD Biosciences, San Jose, CA, USA). This percentage was then multiplied by the WBC concentration measured using 5-part differential hematology analysers (XN-9000; Sysmex, Kobe, Japan). 0th-, 5th-, 10th-, 25th-, 50th-, 75th-, 90th-, 95th- and 100th-percentile values of Abs CD34-positive were 3.9, 18.0, 29.9, 46.9, 72.2, 112.1, 153.0, 190.8 and 501.8 × 10E+5 cells. Corresponding values for CD34-positive/BW were 0.17, 0.60, 0.83, 1.28, 1.90, 2.79, 4.38, 5.79 and 10.90 × 10E+5/kg and for CD34-postive/BV, 0.02, 0.09, 0.12, 0.18, 0.27, 0.40, 0.61, 0.80 and 2.40 × 10E+7/L.

We considered 3 expressions of CD34-positive cell dose: (1) absolute numbers of CD34-positive cells (Abs CD34-positive); (2) number of CD34-positive cells *per* kg of recipient body weight (CD34-positive/BW); and (3) number of CD34-positive cells *per* liter of recipient blood volume (CD34-postive/BV). Recipient blood volume was estimated by multiplying 83.8 ml/kg with lean body mass, which was estimated based on sex, age, body height and body weight [4].

Interval to granulocyte recovery was defined as the date of the 1st of 3 consecutive measurements over ≥ 3 d of a blood granulocyte concentration > 0.5 × 10E+9/L. 10 subjects (2%) died before granulocyte recovery; 609 had granulocyte recovery at a median of 16 d (Range, 10 – 57 d). Cumulative incidences of granulocyte recovery ware 30% (26, 34%), 84% (81, 87%) and 95% (93, 97%) at 14, 21 and 30 d posttransplant. For each measure of CD34-positive cell dose we calculated two concordance values (**Table 2**; **Supplement**

Table 2): (1) concordance with time of granulocyte recovery (equivalent to the area under the receiver-operating characteristic curve) [5] ; and (2) concordance with granulocyte recovery after > 21 days by fitting a logistic regression model [6].

Non-linear proportional hazards model for granulocyte recovery

The model we used to estimate the effect of CD34-positive cell dose on the rate of granulocyte recovery is a Bayesian extension of the Cox regression model with restricted cubic splines [7]. We used roughness penalty minimization [8] to encourage smoothness of the fitted curve and sample an ensemble of curves from the posterior distribution using Markov chain Monte Carlo [9] to estimate the confidence intervals (CIs) of the curve. Our method is similar in spirit but distinct from previous studies that relied on using a small number of spline knots [7], random walk smoothness priors [10] or integrated nested Laplace approximation [11] for curve estimation.

Denote subject's granulocyte recovery time as τ_i . Denote subject's instantaneous event rate at time *t* as $\lambda_i(t)$, which assumes the form of $\lambda_i(t) = \lambda_0(t)\theta(x_i)$, where $\lambda_0(t)$ is the baseline event rate, x_i is subject's CD34-positive cell dose and the "hazard function" $\theta(x)$ is a nonlinear function of *x*. The algorithm assumes proportional hazards across the population, but unlike Cox regression it does not constrain the hazard function to be a straight line.

Assume subjects are ordered according to CD34-positive cell dose: $x_1 \leq \cdots \leq x_N$. The partial likelihood function is $L(\theta) = \prod_{i=1}^{N} \frac{\theta(x_i)}{\sum_{j:\tau_j \geq \tau_i} \theta(x_j)}$. In addition, we assume $f(\cdot) = \log(\theta(\cdot))$ is a natural cubic spline with the constraint

$\sum_{i=1}^{N} f(x_i) = 0$ and prior probability $P(\theta) \propto \exp\left(-\gamma \int_{-\infty}^{\infty} \left(\frac{d^2}{dx^2} f(x)\right)^2 dx\right)$, where $\int_{-\infty}^{\infty} \left(\frac{d^2}{dx^2} f(x)\right)^2 dx$ can be computed exactly using linear algebra [8]. The constraint $\sum_{i=1}^{N} f(x_i) = 0$ forces the geometric mean of the hazard function $\theta(x)$ to be 1 across the study cohort; in other words, hazard is calculated with respect to the population mean. Knots for the spline were chosen at the 5th-, 10th-, 25th-, 50th-, 75th-, 90th- and 95th-percentile values of the study cohort. The intuition behind $P(\theta)$ is to penalise curves that are too "rugged".

Our goal is to maximise the posterior probability $L(\theta)P(\theta)$ by identifying the optimal $\theta(\cdot)$ and also to estimate the variance of $\theta(\cdot)$ around its optimal solution. We achieved this by running a Markov chain Monte Carlo procedure (**Appendix**) that performed the Metropolis-Hastings algorithm [9] . After the burn-in period, 2000 samples were drawn from the posterior distribution to estimate the median curve and the 95-percent CI (**Figure 1**). Estimates of $\theta(\cdot)$ were plotted between the 2.5th- and 97.5th-percentile doses; the computed curve was deemed unreliable outside this range due to insufficient data support.

When the hazard function is calculated as described above, "hazard = 0.5" means that the instantaneous recovery rate (from day 1 posttransplant to infinity) is half-magnitude compared to the population mean. "Hazard = 0" means zero probability of granulocyte recovery.

Modeling the early kinetics of granulocyte recovery

Denote the infused CD34-positive cell dose (CD34-positive/BV; unit: 10E+7/L) as x_0. Assume infused CD34-positive cells are distributed across the entire blood volume of the recipient. Assume after infusion the granulocyte population in blood doubles every δ days. Denote the interval to granulocyte recovery as τ. Then, τ is expected to have a linear relationship with the

logarithm (base 2) of x_0, and the slope is $-\delta$ (**Figure 1**). We can estimate δ by fitting a linear regression model of granulocyte recovery interval *versus* $\log_2$(CD34-positive/BV).

Sensitivity analyses

To exclude potential confounding effects of subjects receiving radiation for pretransplant conditioning (7%) and of methotrexate for posttransplant immune suppression (1%), we repeated our analyses excluding these subjects to assess the robustness of our results (**Supplement Table 2**; **Supplement Figures 1 & 2**).

Follow-up

Posttransplant blood concentrations of natural killer (NK)-, CD8-positive T-, CD4-positive T- and B-cells were available for 447 (72%) subjects, with a total of 1834 samples collected after day 30 posttransplant (median time 187 d (range, 31 – 1984 d) posttransplant; **Supplement Figure 3**). Reference ranges of immune cell concentrations were based on normal subjects [12]. Median follow-up of survivors is 3.1 years (95% CI, [1.4, 6.6]; **Supplement Figure 4**). Relapse was defined as blasts ≥ 5% in bone marrow aspirations, pathological blood blasts or extra-medullary relapse.

Multi-variable Cox regression analysis of granulocyte recovery

To evaluate if CD34-positive/BV is an independent predictor of the rate of granulocyte recovery, we fitted a multi-variate Cox regression model including the following co-variates (**Supplement Figure 5**): transplant year, patient age, sex, disease risk, success/failure of reaching the 1st histological complete remission, conditioning regimen, number of mismatched HLA loci, number of

donor activating killer-cell immunoglobulin-like receptor (KIR) genes, presence/absence of inhibitory KIR ligand mismatch, ABO mismatch, CD34-positive/BV and posttransplant immune suppression regimen. *P* values were adjusted using the Benjamini-Hochberg procedure [13] to correct for multiple variables.

References:

1. Dohner H, Wei AH, Appelbaum FR, Craddock C, DiNardo CD, Dombret H, et al. Diagnosis and management of AML in adults: 2022 recommendations from an international expert panel on behalf of the ELN. Blood. 2022;140(12):1345-77.
2. NCCN clinical practice guidelines in oncology [Available from: https://www.nccn.org/professionals/physician_gls/pdf/all.pdf.
3. Sutherland DR, Anderson L, Keeney M, Nayar R, Chin-Yee I. The ISHAGE guidelines for CD34+ cell determination by flow cytometry. International Society of Hematotherapy and Graft Engineering. J Hematother. 1996;5(3):213-26.
4. Raes A, Van Aken S, Craen M, Donckerwolcke R, Vande Walle J. A reference frame for blood volume in children and adolescents. BMC Pediatr. 2006;6:3.
5. Steyerberg EW, Vickers AJ, Cook NR, Gerds T, Gonen M, Obuchowski N, et al. Assessing the performance of prediction models: a framework for traditional and novel measures. Epidemiology. 2010;21(1):128-38.
6. Austin PC, Steyerberg EW. Interpreting the concordance statistic of a logistic regression model: relation to the variance and odds ratio of a continuous explanatory variable. BMC Med Res Methodol. 2012;12:82.
7. Heinzl H, Kaider A. Gaining more flexibility in Cox proportional hazards regression models with cubic spline functions. Comput Methods Programs Biomed. 1997;54(3):201-8.
8. Green PJ, Silverman BW. Nonparametric Regression and Generalized Linear Models: A Roughness Penalty Approach. London: Chapman & Hall; 1994.
9. Kirkpatrick S, Gelatt CD, Jr., Vecchi MP. Optimization by simulated annealing. Science. 1983;220(4598):671-80.
10. Hennerfeind A, Brezger A, Fahrmeir L. Geoadditive survival models. J Am Stat Assoc. 2006;101:1065-75.
11. Rue H, Martino S. Approximate Bayesian inference for latent Gaussian models by using integrated nested Laplace approximations. J R Statist Soc B. 2009;71:319-92.
12. Wong WS, Lo AW, Siu LP, Leung JN, Tu SP, Tai SW, et al. Reference ranges for lymphocyte subsets among healthy Hong Kong Chinese adults by single-platform flow cytometry. Clin Vaccine Immunol. 2013;20(4):602-6.
13. Benjamini Y, Hochberg Y. Controlling the false discovery rate: a practical and powerful approach to multiple testing. J R Statist Soc B. 1995;57(1):289-300.

Supplement Table 1. Subject co-variates (*N* = 619).

**Year at transplant, n (%)**	
2015	25 (4)
2016	51 (8)
2017	109 (18)
2018	132 (21)
2019	163 (26)
2020	139 (23)
**Age, years, median (range)**	13.6 (0.7 – 62.3)
**Male, n (%)**	343 (55)
**Height cm, median (range)**	155.0 (72.0 – 187.0)
**Weight kg, median (range)**	44.5 (8.0 – 102.0)
**Leukemia, n (%)**	
Low-risk ALL	123 (20)
High-risk ALL	147 (24)
Standard-risk AML	77 (12)
Intermediate-risk AML	174 (28)
High-risk AML	88 (14)
MPAL	10 (2)
**Leukaemia state, n (%)**	
CR1	352 (57)
Not CR1	267 (43)
**Pretransplant conditioning, n (%)**	
Radiation + CY + others	43 (7)
BU + CY + others	576 (93)
**HLA mismatch in G*v*HD direction, n (%)**	
None	31 (5)
1 locus	72 (12)
2 loci	170 (28)
3 loci	188 (30)
4 loci	136 (22)
5 loci	22 (4)
**Inhibitory KIR ligand mismatch in G*v*HD direction, n (%)**	119 (19)
**N donor activating KIR genes, n (%)**	
1	306 (49)
2	29 (5)
3	36 (6)
4	183 (30)
5	47 (8)
6	18 (3)
**ABO, n (%)**	
Match	204 (33)

Major mis-match	165 (27)
Minor mis-match	170 (28)
Bi-directional mis-match	80 (13)
**Absolute CD34-positive cell dose, 10E+5 cells, median (range)**	72.15 (3.85 – 501.75)
**CD34-positive/BW cell dose, 10E+5 cells/kg, median (range)**	1.90 (0.17 – 10.90)
**CD34-positive/BV cell dose, 10E+7 cells/L, median (range)**	0.27 (0.02 – 2.40)
**Posttransplant immune suppression, n (%)**	
CSA + MMF	612 (99)
CSA + MMF + MTX	7 (1)

Abbreviations: AML, acute myeloid leukaemia; ALL, acute lymphoblastic leukaemia; BU, Busulfan; BV, blood volume; BW, body weight; CR, complete remission; CSA, cyclosporine; CY, cyclophosphamide; KIR, killer-cell immunoglobulin-like receptor; MMF, mycophenolate mofetil; MPAL, mixed phenotype acute leukaemia; MTX, methotrexate; G*v*HD, graft-*versus*-host disease.

Supplement Table 2. Concordance of infused CD34-positive cell dose with granulocyte recovery when the subjects receiving pretransplant radiation and/or posttransplant methotrexate were censored.

Patient selection flowchart is displayed in Supplement Figure 1.

	N	**Absolute CD34-positive**	**CD34-positive per BW**	**CD34-positive per BV**
**Concordance with interval to recovery**				
All cases	569	0.558	0.606	0.609
"Extreme" LBM/BW ratio[a]	173	0.533	0.599	0.608
"Normal" LBM/BW ratio[a]	396	0.568	0.612	0.613
**Concordance with successful recovery by d 21 posttransplant[b]**				
All cases	560	0.620	0.638	0.658
"Extreme" LBM/BW ratio[a]	171	0.565	0.585	0.630
"Normal" LBM/BW ratio[a]	389	0.637	0.680	0.686

Abbreviations: BV, blood volume; BW, body weight; LBM, lean body mass.

[a] "Extreme", < 15th- or > 85th-percentile values; "normal", other cases.

[b] Patients who died before granulocyte recovery were excluded from this analysis.

Supplement Figure 1. Patient selection flowchart.

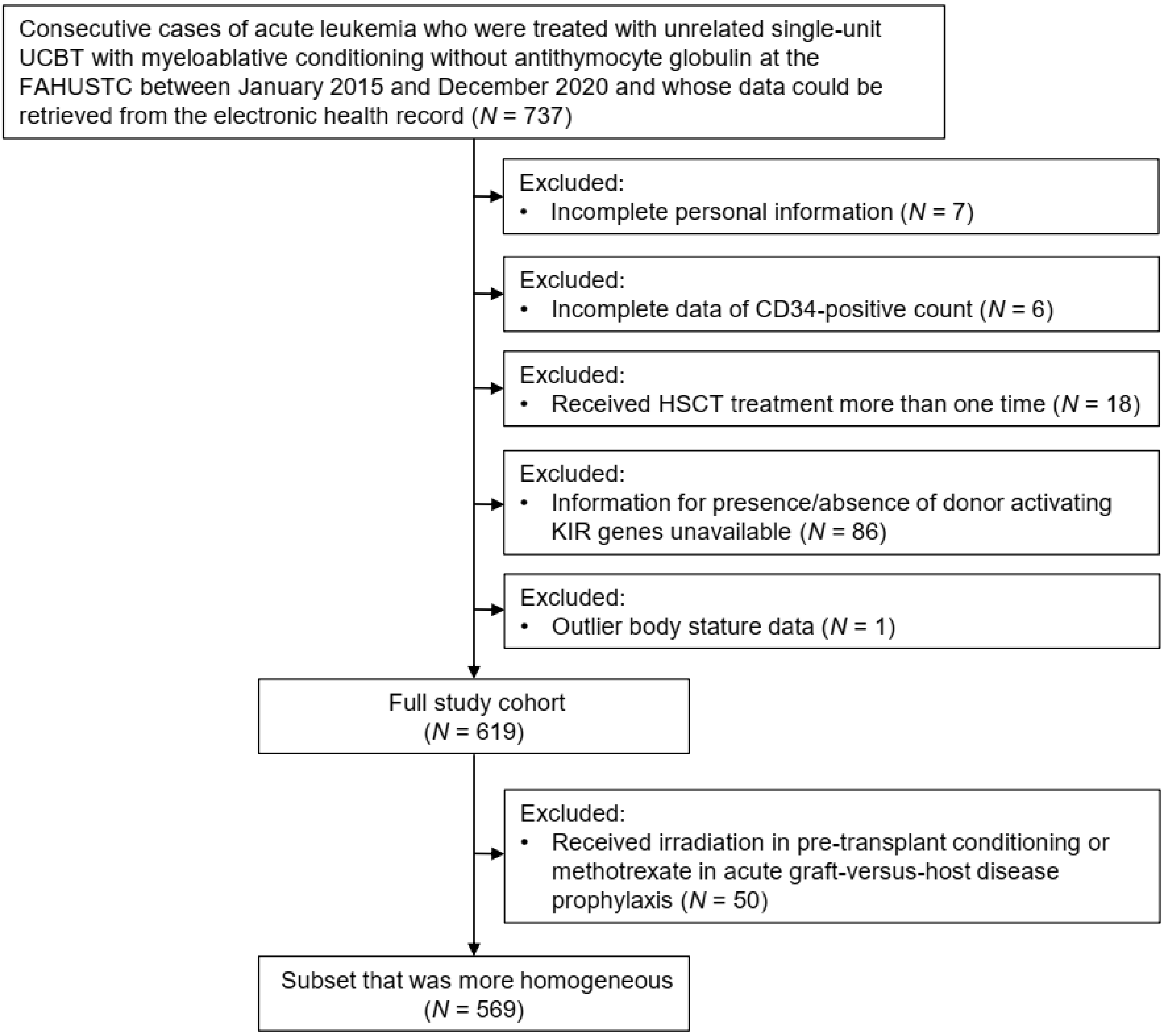

Supplement Figure 2. Analytical results when the subjects receiving pretransplant radiation and/or posttransplant methotrexate were censored.

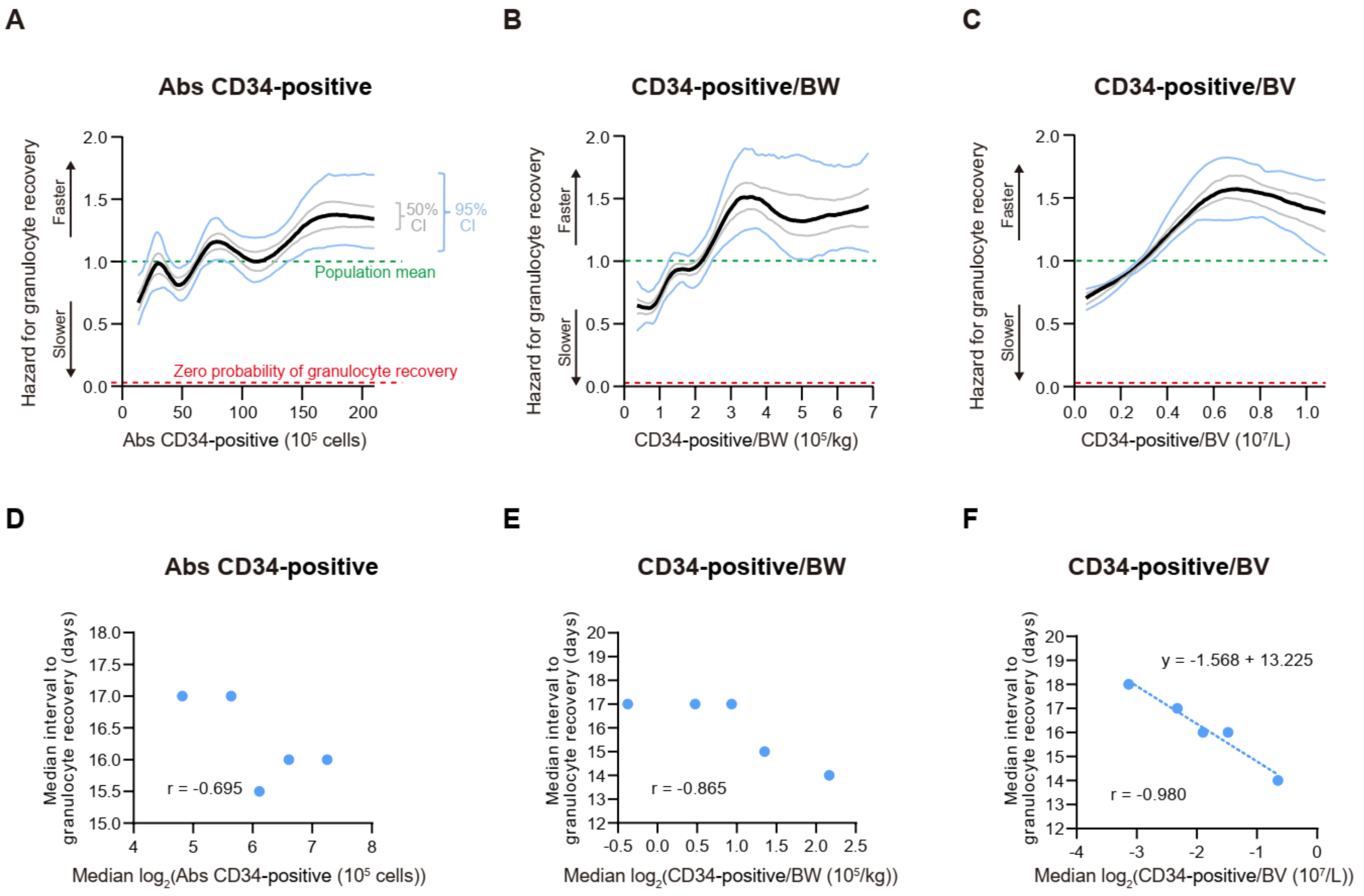

Patient selection flowchart is displayed in Supplement Figure 1.

A, B, C Hazard functions of Abs CD34-positive (**A**), CD34-positive/BW (**B**) and CD34-positive/BV (**C**) for granulocyte recovery (*N* = 569).

D, E, F Relationship between CD34-positive cell dose and interval to granulocyte recovery interval: Abs CD34-positive (**D**), CD34-positive/BW (**E**) and CD34-positive/BV (**F**) (*N* = 560; patients who died before granulocyte recovery were excluded from this analysis).

Abbreviations: Abs, absolute; BV, blood volume; BW, body weight.

Supplement Figure 3. Recovery of immune cell counts in blood at different levels of infused CD34-positive cell dose per blood volume.

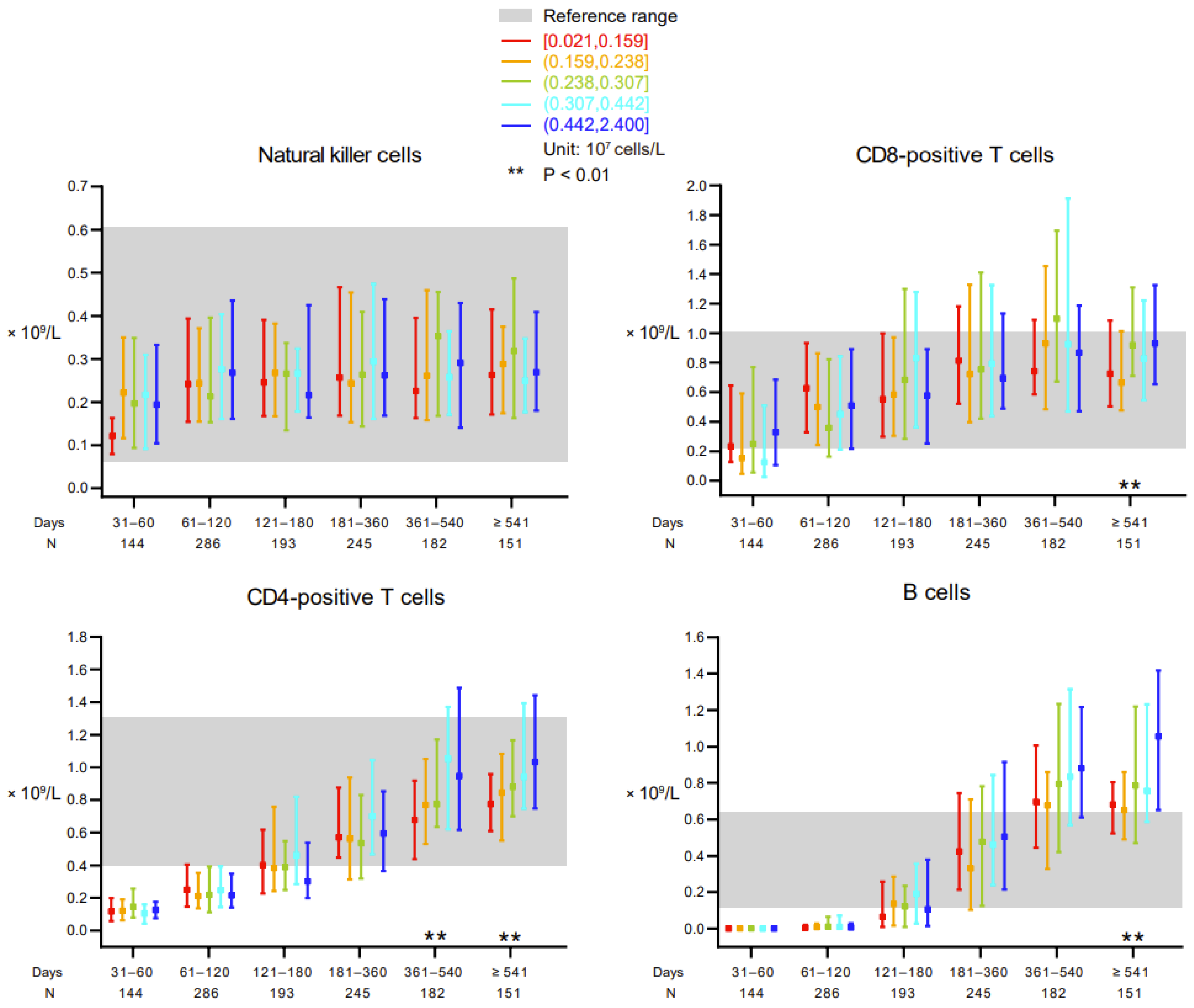

The study cohort is divided into 5 quintiles according to CD34-postive/BV. N denotes the number of patients that had available data for peripheral blood immune cell counts within a time interval. (**, $P < 0.01$ [Kruskal-Wallis])

Supplement Figure 4. Clinical outcomes at different levels of infused CD34-positive cell dose per blood volume (*N* = 619).

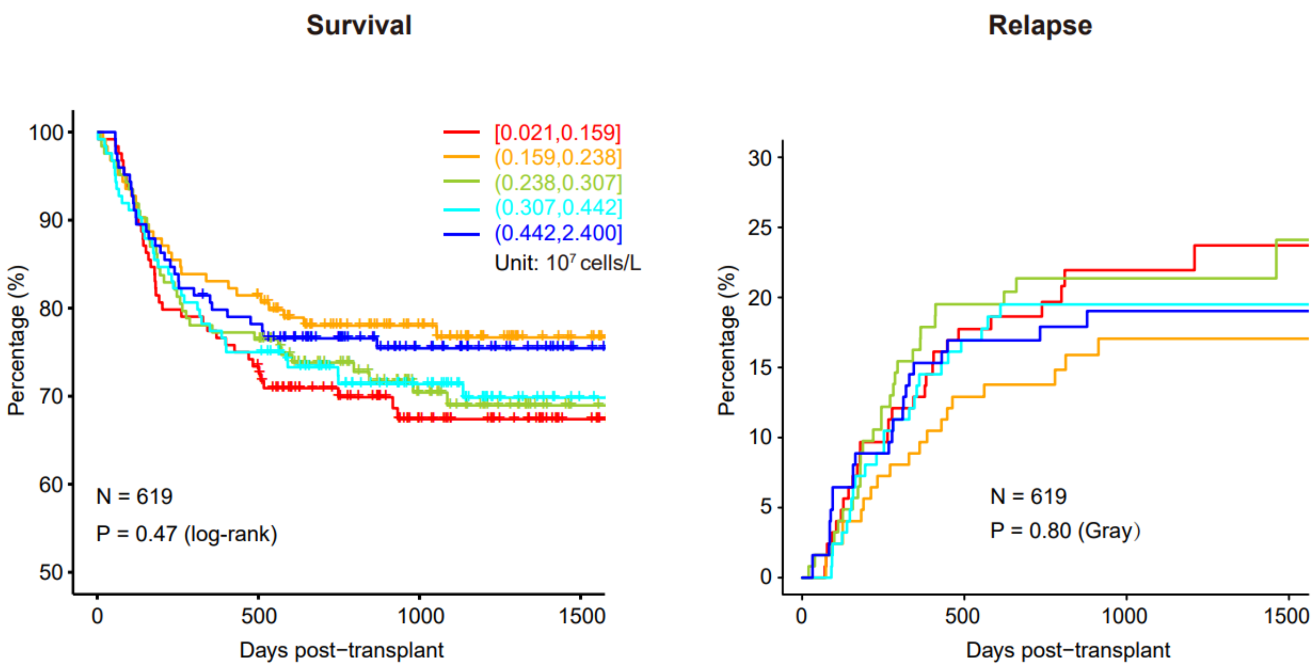

The study cohort is divided into 5 quintiles according to CD34-positive/BV.

Supplement Figure 5. Multi-variate Cox regression analysis of granulocyte recovery (*N* = 619).

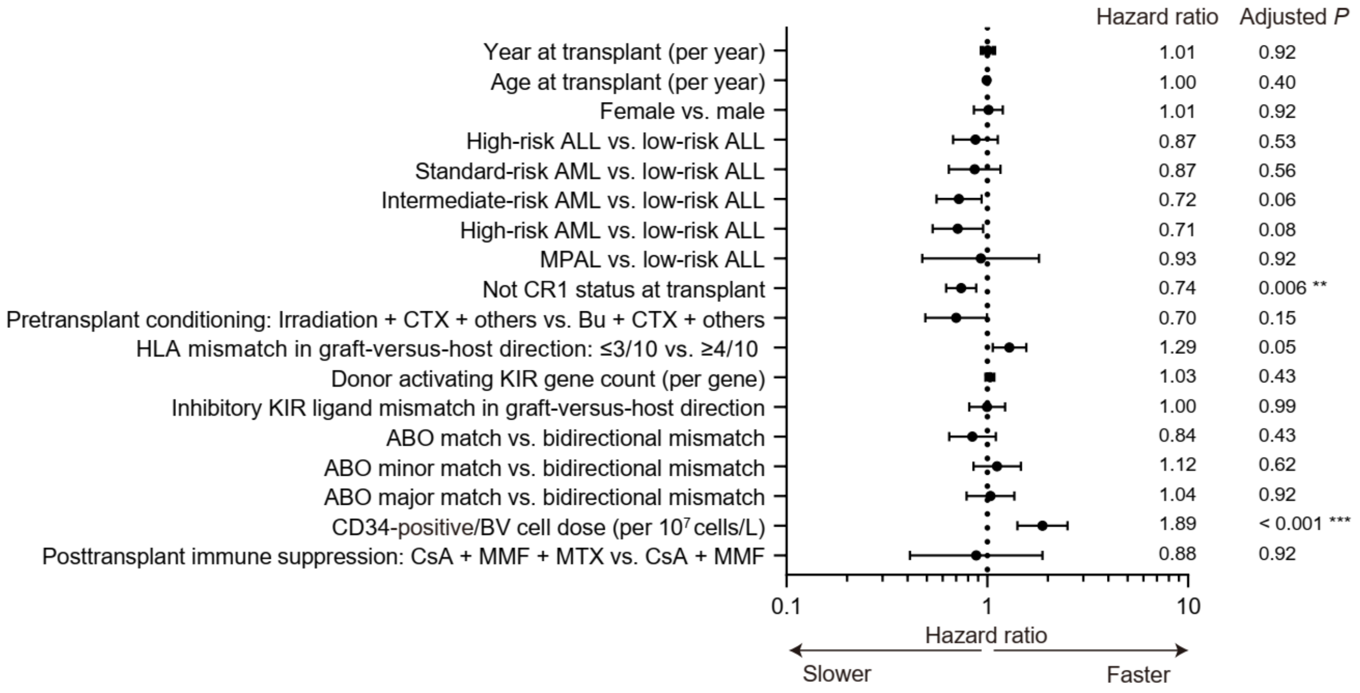

(**, adjusted P < 0.01; ***, adjusted P < 0.001)

Appendix

Here we provide a step-by-step explanation of the algorithm:

Step 1. Compute $h_i \leftarrow x^{(i+1)} - x^{(i)}$ $(i = 1, \cdots, 6)$.

Step 2. Compute a 5×7 matrix $\mathbf{D}$ as follows:

$D_{i,i} \leftarrow h_i^{-1}$ $(i = 1, \cdots, 5)$

$D_{i,i+1} \leftarrow -h_i^{-1} - h_{i+1}^{-1}$ $(i = 1, \cdots, 5)$

$D_{i,i+2} \leftarrow h_{i+1}^{-1}$ $(i = 1, \cdots, 5)$

(All the other elements of $\mathbf{D}$ are zero.)

Step 3. Compute a 5×5 matrix $\mathbf{W}$ as follows:

$W_{i,i} \leftarrow \frac{h_i + h_{i+1}}{3}$ $(i = 1, \cdots, 5)$

$W_{i,i-1} \leftarrow \frac{h_i}{6}$ $(i = 2, \cdots, 5)$

$W_{i-1,i} \leftarrow \frac{h_i}{6}$ $(i = 2, \cdots, 5)$

(All the other elements of $\mathbf{W}$ are zero.)

Step 4. Compute a 7×7 matrix $\mathbf{A}$ as follows:

$\mathbf{A} \leftarrow \mathbf{D}^{\mathrm{T}}\mathbf{W}^{-1}\mathbf{D}$

Step 5. Recall that $f(\cdot)$ is a restricted cubic spline with knots at $x^{(1)}, \cdots, x^{(7)}$.

Denote $g^{(i)} = f\left(x^{(i)}\right)$ $(i = 1, \cdots, 7)$ and $\boldsymbol{g} = \left(g^{(1)}, \cdots, g^{(7)}\right)^T$. Define a function $\Psi(\boldsymbol{g})$ that computes the penalised partial log-likelihood as follows:

$\Psi \leftarrow$ function($\boldsymbol{g}$){

$\varphi \leftarrow \sum_{i=1}^{N} f(x_i)$

For $(t = 1, \cdots, \max_i \tau_i)$ {

$\mathbb{C}^{(t)} \leftarrow \{i\colon \tau_i \geq t\}$

$\aleph^{(t)} \leftarrow \{i:\ \tau_i = t\}$

While $(|\aleph^{(t)}| > 0)$ {

$$\varphi \leftarrow \varphi - \log \sum_{j \in \mathbb{C}^{(t)}} \exp\left(f(x_j)\right)$$

Pick one patient *i* from $\aleph^{(t)}$

$\aleph^{(t)} \leftarrow \aleph^{(t)} - \{i\}$

$\mathbb{C}^{(t)} \leftarrow \mathbb{C}^{(t)} - \{i\}$

}

}

$\varphi \leftarrow \varphi - \gamma \boldsymbol{g}^{\mathrm{T}} \mathbf{A} \boldsymbol{g}$

Return φ

}

Step 6. Iterate the following Markov chain to equilibrium and then sample 2000 $\boldsymbol{g}$'s from the posterior distribution by running additional 2000 iterations:

$\boldsymbol{g} \leftarrow (0, \cdots, 0)^T$

Iterate {

$\boldsymbol{g}_{\text{proposed}} \leftarrow \boldsymbol{g}$

Randomly select *k* from $\{1, \cdots, 7\}$

Draw a random number $\rho \sim \text{Uniform}(-2, 2)$

$g^{(k)}_{\text{proposed}} \leftarrow \rho$

Draw a random number $\rho \sim \text{Uniform}(0, 1)$

If $\rho < \exp\left(\Psi(\boldsymbol{g}_{\text{proposed}}) - \Psi(\boldsymbol{g})\right)$ then {

$\boldsymbol{g} \leftarrow \boldsymbol{g}_{\text{proposed}}$

$\mu \leftarrow \sum_{i=1}^{N} f(x_i)/N$

$\boldsymbol{g} \leftarrow \boldsymbol{g} - \mu(1, \cdots, 1)^T$

}

}
